# Supplementary material for: The causal relationship between the human gut microbiota and pyogenic arthritis: a Mendelian randomization study
Source: Front Cell Infect Microbiol. 2024 Nov 26;14:1452480. doi: 10.3389/fcimb.2024.1452480 (PMC11629706; doi:10.3389/fcimb.2024.1452480)
Supplement: Supplementary file 1 [file DataSheet1.zip › mendelian test/ebi-a-GCST90027553.csv_leave_one_out.pdf]

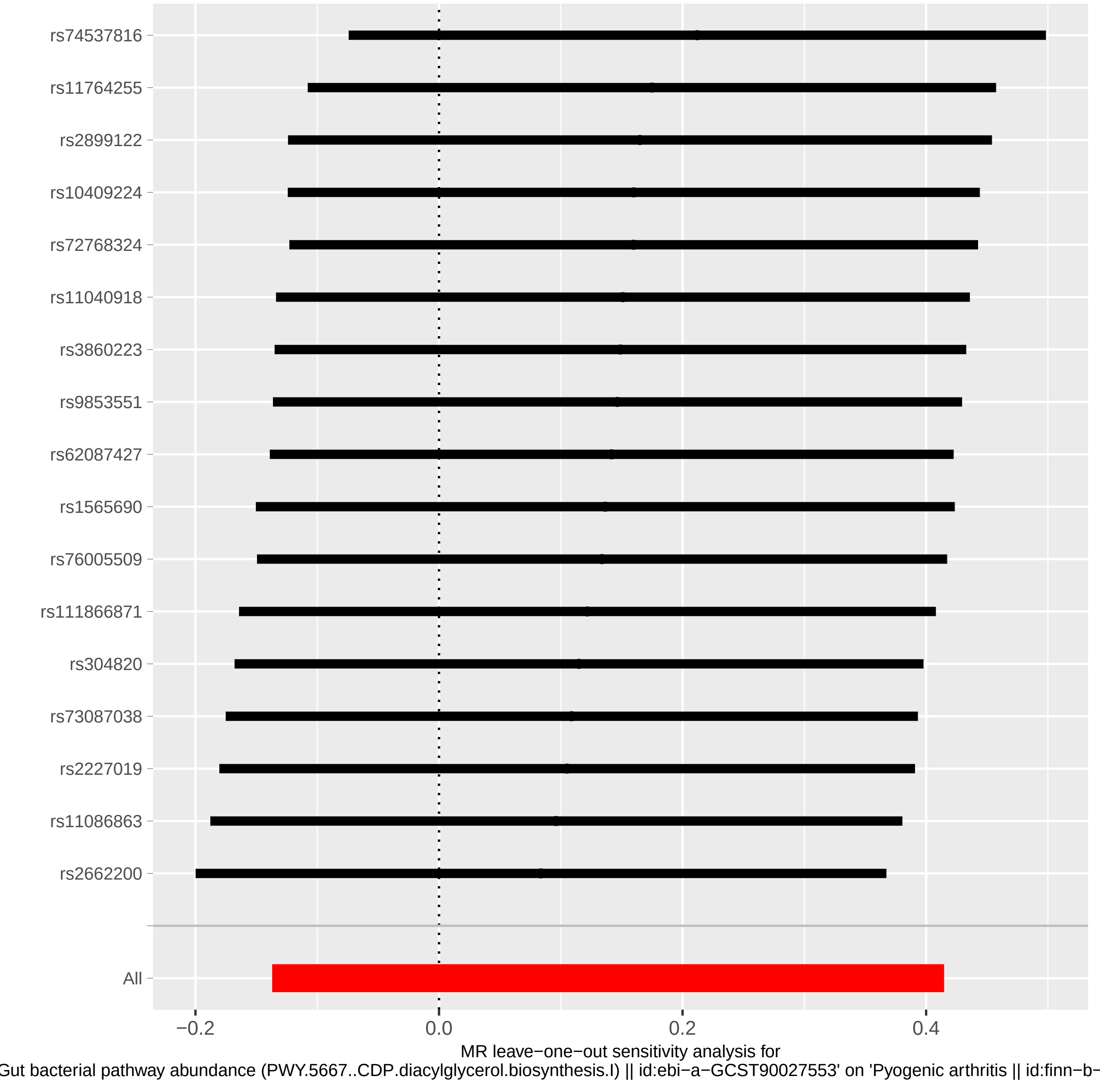
rs74537816
rs11764255
rs2899122
rs10409224
rs72768324
rs11040918
rs3860223
rs9853551
rs62087427
rs1565690
rs76005509
rs111866871
rs304820
rs73087038
rs2227019
rs11086863
rs2662200
All
−0.2
0.0
0.2
0.4
MR leave−one−out sensitivity analysis for
'Gut bacterial pathway abundance (PWY.5667..CDP.diacylglycerol.biosynthesis.I) || id:ebi−a−GCST90027553' on 'Pyogenic arthritis || id:finn−b−
